# Fixation Methods for Mandibular Advancement and Their Effects on Temporomandibular Joint: A Finite Element Analysis Study

**DOI:** 10.1155/2020/2810763

**Published:** 2020-02-21

**Authors:** Sabit Demircan, Erdoğan Utku Uretürk, Ayşegül Apaydın, Sinan Şen

**Affiliations:** ^1^Beykent University Vocational School Dental Services, Oral Health Program, Istanbul, Turkey; ^2^Istanbul University Faculty of Dentistry Department of Oral and Maxillofacial Surgery, Istanbul, Turkey; ^3^Department of Orthodontics and Dentofacial Orthopaedics, University of Heidelberg, Heidelberg, Germany

## Abstract

**Objectives:**

Bilateral sagittal split osteotomy (BSSO) is a common surgical procedure to correct dentofacial deformities that involve the mandible. Usually bicortical bone fixation screw or miniplates with monocortical bone fixation screw were used to gain stability after BSSO. On the other hand, the use of resorbable screw materials had been reported. In this study, our aim is to determine first stress distribution values at the temporomandibular joint (TMJ) and second displacement amounts of each mandibular bone segment.

**Methods:**

A three-dimensional virtual mesh model of the mandible was constructed. Then, BSSO with 9 mm advancement was simulated using the finite element model (FEM). Fixation between each mandibular segment was also virtually performed using seven different combinations of fixation materials, as follows: miniplate only (M), miniplate and a titanium bicortical bone fixation screw (H), miniplate and a resorbable bicortical bone fixation screw (HR), 3 L-shaped titanium bicortical bone fixation screws (L), 3 L-shaped resorbable bicortical bone fixation screws (LR), 3 inverted L-shaped titanium bicortical bone fixation screws (IL), and 3 inverted L-shaped resorbable bicortical bone fixation screws (ILR).

**Results:**

At 9 mm advancement, the biggest stress values at the anterior area TMJ was seen at M fixation and LR fixation at posterior TMJ. The minimum stress values on anterior TMJ were seen at L fixation and M fixation at posterior TMJ. Minimum displacement was seen in IL method. It was followed by L, H, HR, M, ILR, and LR, respectively.

**Conclusion:**

According to our results, bicortical screw fixation was associated with more stress on the condyle. In terms of total stress value, especially LR and ILR lead to higher amounts.

## 1. Introduction

Bilateral sagittal split osteotomy (BSSO) is a widely performed surgical approach among orthognathic surgery methods for the treatment of mandibular discrepancies. Since the first original description by Trauner and Obwegeser, various modifications of this method, e.g., by Dal Pont, Epker, and Hunsuck, have been proposed and contributed to substantial progress in orthognathic surgery. Through the use of the modern metal plates and screws after osteotomy, the stability can be already achieved in a technique so-called “rigid internal fixation” (RIF), without using “intermaxillary fixation” (IMF). The introduction of rigid internal fixation devices such as miniplates and screws showed increased application and acceptance of orthognathic surgery, because they are compliance-independent approaches to stabilize the mandibular segments after BSSO. RIF methods contribute to postoperative bone healing and masticatory function. Furthermore, using RIF method, instead of intermaxillary rigid fixation, can initiate the early improvement of oral hygiene [1–4].

In spite of the advantages of RIF method shown by various studies, there are still controversies regarding the alterations in condylar position after BSSO. The stress distribution in the temporomandibular joint (TMJ) can lead to malocclusion, early relapse, and also risk of temporomandibular disorders (TMD). Thus, several navigation devices have been proposed and applied for intraoperative condylar positioning; however, there are no better long-term benefits in BSSO [5]. Only very few studies investigated the impact of BSSO on the TMJ [6, 7]. Ureturk and Apaydin showed using a finite element model (FEM) of a mandible that different forces occurred on the TMJ depending on different RIF techniques by a mandibular advancement of 5 mm [3].

Another possible clinically relevant postoperative outcome is postoperative skeletal stability, which might be dependent on the choice of fixation instruments, such as bicortical and monocortical fixation and resorbable materials. Al-Moraissi and Al-Hendi showed in their systematic review and meta-analysis no clinically relevant difference in postoperative skeletal stability between monocortical plate and bicortical fixation screw. Nevertheless, the meta-analysis was performed on three clinical studies, only one of the studies included was a randomized controlled trial. Based on this finding, there is a consensus that the amount of advancement is directly proportional to the amount of relapse [8]. However, there are more RIF materials such as biological inert or resorbable materials or materials with different geometrical design, which might lead other stress distribution on the mandibular segments after fixation [5].

Thus, maintaining condylar position and obtaining the stability without influencing a relapse of mandibular segments using different RIF techniques and materials still remain to be the focus of further evaluations. The outcomes might help surgeons on the selection of the different RIF approaches. The aim of this present study was to evaluate two clinically relevant outcomes by simulating a mandibular advancement of 9 mm using FEM of the mandible: (i) stress distribution values at the areas of the temporomandibular joint (TMJ) and (ii) displacement amounts of each mandibular bone segment.

## 2. Materials and Methods

### 2.1. Preparation of a 3-Dimensional Mandible Model via Cone Beam Computer Tomography

**Figure 1:**
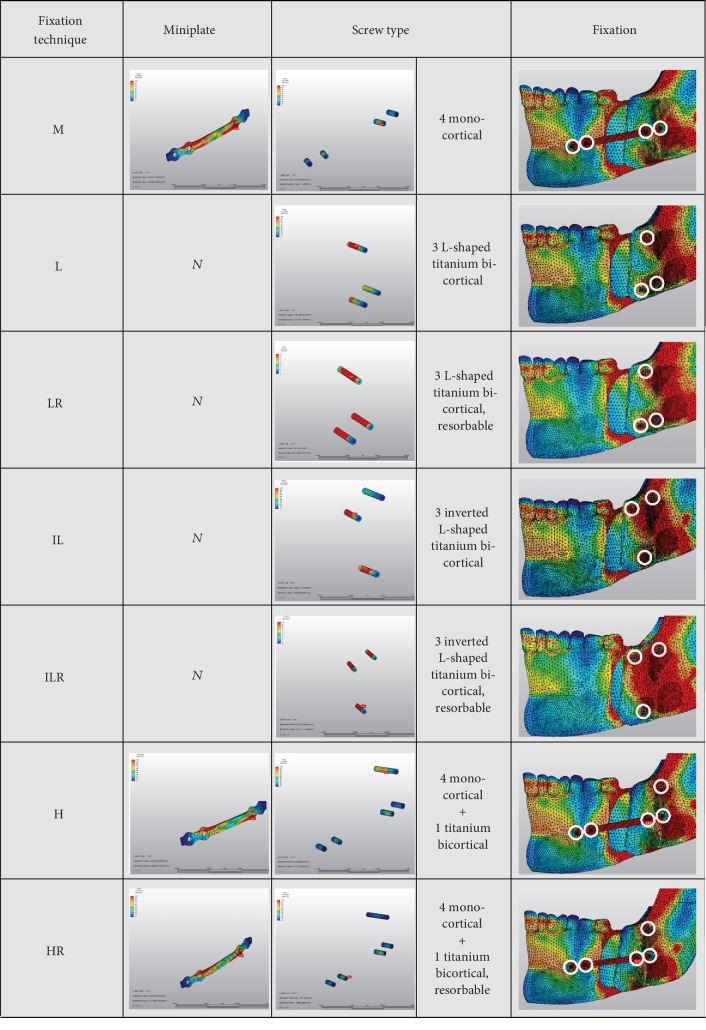
Illustrations of seven different fixation techniques used in this study. Rightmost panel: white rings show the locations of the insertion areas of the screws.

Cone beam computer tomography (CBCT) imaging was performed using Galileos Comfort Plus (Sirona Dental Systems, Germany). The following 3D X-ray imaging parameters have been set: 98 kVp/6 mA and 0.5 mm slice thickness. Based on the DICOM data output, the 3D voxel mesh mandible model was generated by VRMesh Studio (VirtualGrid Inc., USA). The resulting 3D mesh model compounding the peripheral cortical zone and the central cancellous zone of the mandible, condyles with their boundary condition, and the fixation materials was subjected to the basic mechanical property set of involved elements according to the established FEM of Ureturk and Apaydin [3]. The modified BSSO by Obwegeser-Dal Pont with 9 mm mandibular advancement was performed, and fixations of the mandibular segments were done with seven different options (Table 1). To illustrate the seven fixation options mentioned above, they are shown in Figure 1.

The positioning of miniplates was performed regarding Champy et al.'s geometries [9]. The inferior alveolar nerve and the roots carefully considered while positioning the bicortical bone fixation screws and screws positioned as far as each other. At the techniques with bicortical screws, the positioning of screws was selected on the superior-posterior of the plates as described in similar studies [10, 11]. Performed occlusal loads and directions given at Tables 2 and 3. Stress distribution of the condyle and fixation devices was assessed using Algor Fempro software (Algor Inc., USA).

After the forces were applied in these analyses, the amount of displacement of the anterior (mesial) and posterior (distal) bone fragments at the superior-anterior, superior-posterior, inferior-anterior, and inferior-posterior corner points was examined.

### 2.2. Statistical Analyses

The analysis of the relationship among the stress values at the posterior area of TMJ and amount of displacement of mandibular bone segments were performed using Pearson's correlation coefficient and stepwise multiple regression. The statistical analysis was conducted using SPSS 25.0 (IBM Corp., Armonk, NY, USA).

## 3. Results

### 3.1. Stress Distribution

**Figure 2:**
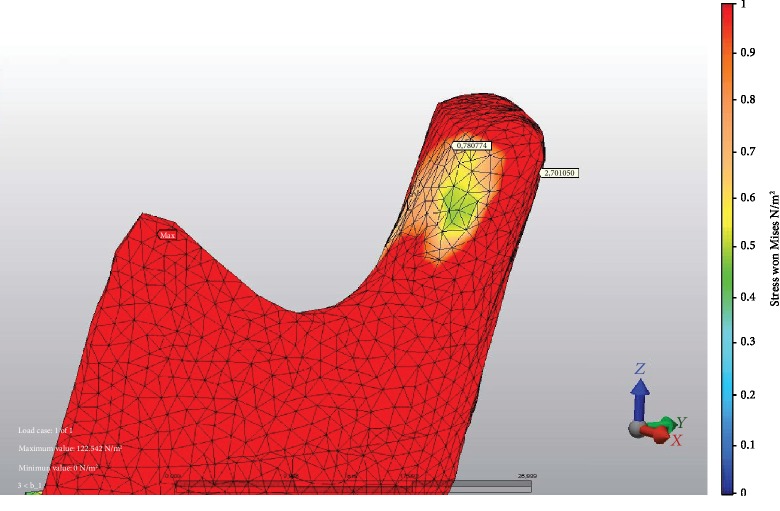
Example of fixation method with the highest stress value recorded at the posterior area of TMJ: LR fixation and stress values at both areas of TMJ.

**Figure 3:**
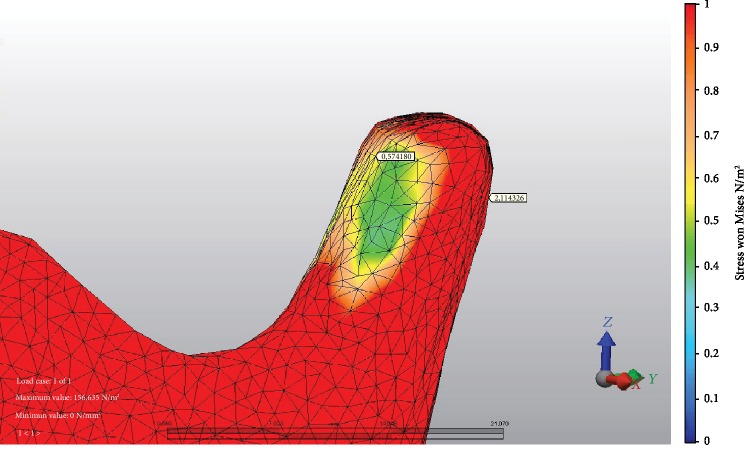
Example of fixation method with the lowest stress value recorded at the anterior area of TMJ: L fixation and stress values at both areas of TMJ.

**Figure 4:**
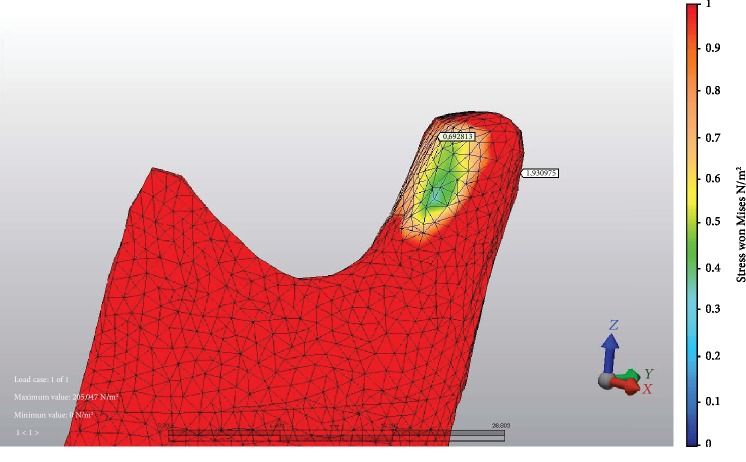
Example of fixation method with the greatest stress value ratio of posterior to anterior area of TMJ: IL fixation and stress values at both areas of TMJ.

At 9 mm advancement, the highest stress values on anterior TMJ were seen at M fixation (Figure 2) and LR fixation at posterior TMJ. The minimum stress values at the anterior area of TMJ were seen at L fixation (Figure 3) and M fixation at the posterior area of TMJ. At miniplate fixations (M, H, and HR), the stress value ratios of posterior to anterior of TMJ were 1.2-1.8-fold, but at bicortical screw fixations (L, LR, IL, and ILR), posterior TMJ stress values were at more than two times higher than anterior TMJ stress values (Figure 4). The minimum stress amount at the posterior region of TMJ was recorded in M fixation and followed by the H, IL, HR, L, ILR, and LR fixations, respectively (Table 4).

### 3.2. Displacement Amounts

Minimum displacement was seen in IL method. It was followed by L, H, HR, M, ILR, and LR, respectively. The biggest displacement on the distal bone segment was seen on LR. The total displacement at this technique was 1.5 times more than IL technique which had the least movement.

The biggest displacement on the mesial bone segment was seen on LR. The total displacement at this technique was 1.5 times more than IL technique which had the least movement (Table 5).

### 3.3. There Was No Correlation between Total Stress Values at TMJ and Amounts of Displacements of Mandibular Segments

**Figure 5:**
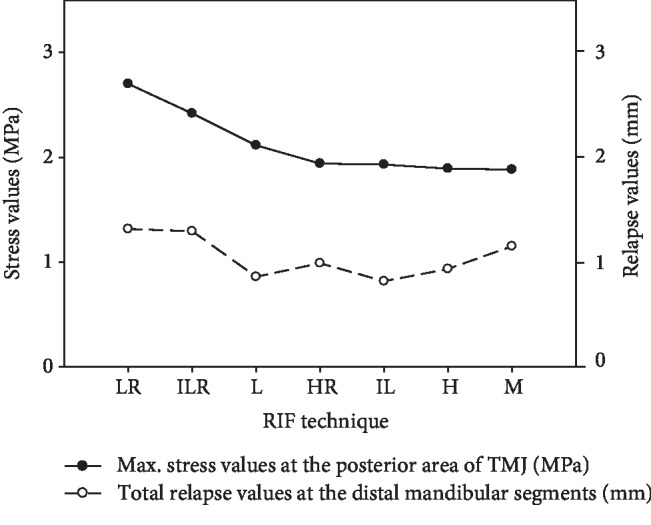
Exemplary comparison of the maximum stress values with total relapse values at the mandibular segments.

In general, the maximum stress values at the TMJ and mandibular segments occurred at the posterior area of the TMJ and distal mandibular segment. There is no correlation between these two outcomes. Overall, the LR technique showed the highest stress variability in terms of total stress value at the posterior area of TMJ, followed by ILR, L, HR, IL, H, and M (Figure 5).

In general, the highest total stress value was recorded at the posterior side of TMJ and the highest total relapse of mandibular segments was found in the posterior (distal) mandibular segment. There is no correlation between these two outcomes. In the figure, the fixation methods were presented in descending order with their maximum stress distribution at TMJ. Therefore, the highest stress value at the TMJ occurred in the LR fixation technique, followed by ILR, L, HR, IL, H, and M.

## 4. Discussion

Relation of TMJ and orthognathic surgery is a controversial topic. The literature has a lot of studies on the calculated relapse values at the mandibular segments, but the present study concentrated on TMJ stress values, which has not been reported previously to the best of the authors' knowledge. Changes in condyles after orthognathic surgery procedures were discussed in many literatures, because these changes can lead the relapse or resorption of the condyle. Fixation devices have a major role in these complications. If the fixation technique leads the stress around the condyle, condylar resorption can cause pain, malocclusion, and TMJ dysfunctions [12].

Chen et al.'s study in 2013 reported that condylar position remained stable 1 year after advancement surgery and changes in condylar position did not increase TMD signs [13]. They concluded that condyle position was more posterior-superior. In such studies, the same position changes of the condyle were reported after BSSO with mandibular advancement [14–16]. Our study results also showed more stress on the posterior part of the condyle with all fixation devices, and these results can be explained with this movement of the condyle.

The hybrid technique was suggested by Schwartz and Relle to enhance benefits of bicortical bone fixation screws and the miniplates with monocortical screws [17]. Our results showed the minimum stress values of the posterior part of the condyle with the miniplate with monocortical bone fixation screws and a titanium bicortical screw (H). Sato et al. reported that with insertion of the bicortical screw there is torsion at the condyles and using the hybrid technique the advantage of the miniplates could be lost. But researchers also reported that the use of a bicortical screw with a miniplate with monocortical screws will be proper to eliminate intercondylar widening at big advancement cases [18].

Hackney et al. investigated the changes in the intercondylar angle and intercondylar width mandibular advancement cases using rigid fixation [19]. Researchers suggest that screw osteosynthesis does not significantly change condylar width or angle and did not cause significant increase in TM symptoms. But in an animal study done by Ellis and Hinton, it was shown that condyle posterior displacement caused resorption of the posterior area of the condyle and anterior zone of the postglenoid spine [20].

Arnett suggested that after BSSO with mandibular advancement cases, the mediolateral torqueing or immediate posterior shift of the condyles after rigid fixation might be dependent on altered loading in the joint for condylar resorption and late relapse [21].

Finally, we showed in our FEM that the highest stress levels at TMJ occurs on the posterior side, where also the highly innervated and vascularized intermediate region of retrodiscal tissue is [22]. These additional loadings might be in the tolerance range of biomechanical properties of the TMJ region [23]. We showed that fixation-related primary relapse of the mandibular segments does not correlate with the stress levels of TMJ. Thus, the predictability of loading on TMJ based on the amounts of fixation-related primary relapse is not applicable. For this reason, we believe that calculation of loadings on TMJ using FEM is in order to avoid possible consequences such as temporomandibular pain, condylar resorption, and late relapse, which is of great clinical relevance.

There are some limitations to this FEM study, because this model was based on anatomical information of an individual case. Nevertheless, there is great progress in computer-aided patient specific orthognathic surgery [24–26]. The FEM analysis presented in this study could also be served prospectively as a tool to plan an orthognathic surgery in a predictable way regarding the stress formation at the surrounding bone compartments [27]. We believe that the stress formation acting on the TMJ is important for long-term functional stability, which should be investigated in further clinical trials.

## 5. Conclusion

Clinicians must always be aware that altered loading on TMJ may cause condylar resorption and late relapse after mandibular advancement cases.

1. According to our results, bicortical screw fixation is associated with more stress on the condyle
2. Taken together, the total stress value on TMJ and relapse amounts and LR and ILR lead to higher values

## Figures and Tables

**Table 1:** Seven different fixation techniques simulated in this FEM study.

Description of technique	Abbreviation
4-hole miniplate with four monocortical bone fixation screws	M
3 L-shaped titanium bicortical bone fixation screws	L
3 L-shaped resorbable bicortical bone fixation screws	LR
3 inverted L-shaped titanium bicortical bone fixation screws	IL
3 inverted L-shaped resorbable bicortical bone fixation screws	ILR
4-hole miniplate with four monocortical screws and a titanium bicortical screw	H
4-hole miniplate with four monocortical screws and a resorbable bicortical screw	HR

**Table 2:** Directions of muscular forces (cos). The resulting 3D mesh model was subjected to the basic mechanical property set of involved elements according to the established FEM of Ureturk and Apaydin, and therefore, the table was reproduced from this previous work [3].

	Directions of muscular forces (cos)		
Muscles	*X*	*Y*	*Z*
Superficial masseter	0.2	0.88	0.41
Deep masseter	0.54	0.75	0.35
Medial pterygoid	0.48	0.79	0.37
Anterior temporalis	0.14	0.98	0.04
Medial temporalis	0.22	0.83	0.5
Posterior temporalis	0.2	0.47	0.85
Superior lateral pterygoid	0.76	0.07	0.64
Anterior digastric	0.24	0.23	0.94

**Table 3:** Dataset of 3-dimensional muscular force application. The resulting 3D mesh model was subjected to the basic mechanical property set of involved elements according to the established FEM of Ureturk and Apaydin, and therefore, the table was reproduced from this previous work [3].

		3D force application		
Muscles	Total force (*N*)	*F*_*x*_ (*N*)	*F*_*y*_ (*N*)	*F*_*z*_ (*N*)
Superficial masseter	190.4	79.7	39.4	163.3
Deep masseter	81.6	29.2	44.5	61.8
Medial pterygoid	174.8	65.2	84.9	138.2
Anterior temporalis	158.0	-6.9	23.5	156.1
Medial temporalis	95.6	47.8	21.2	80.0
Posterior temporalis	75.6	64.6	15.7	35.8
Superior lateral pterygoid	28.7	18.5	21.8	2.1
Anterior digastric	40.0	37.6	9.7	-9.4

**Table 4:** Stress distribution values (MPa) at different areas of TMJ.

TMJ	BSSO with 9 mm mandibular advancement	
	Anterior	Posterior
M	1,484563	1,883807
L	0,574180	2,114326
LR	0,780774	2,701050
IL	0,692813	1,930975
ILR	1,122380	2,418921
H	1,053339	1,892166
HR	1,096860	1,941026

**Table 5:** Displacements (mm) of mesial (Me) and distal (Di) mandibular segments at superior-anterior, superior-posterior, inferior-anterior, and inferior-posterior corners at all fixation techniques on 9 mm advancement.

			M	L	LR	IL	ILR	H	HR
SA	Me	Total	0.013066	0.024300	0.024112	0.008777	0.008431	0.020806	0.013511
	Di	Total	0.31712	0.16444	0.26732	0.13797	0.27539	0.20326	0.22619

SP	Me	Total	0.131441	0.169985	0.196611	0.138070	0.172643	0.209712	0.201064
	Di	Total	0.334327	0.190981	0.279554	0.204063	0.309638	0.251797	0.264078

IA	Me	Total	0.057080	0.052055	0.049330	0.052779	0.049324	0.056355	0.056947
	Di	Total	0.17960	0.14376	0.21359	0.13832	0.10792	0.16158	0.16801

IP	Me	Total	0.159397	0.203939	0.224159	0.188539	0.200973	0.182714	0.175584
	Di	Total	0.319511	0.359756	0.554576	0.336255	0.600206	0.317153	0.330171

Mesial		Total	0.360984	0.450279	0.494212	0.388165	0.431371	0.469587	0.447106

Distal		Total	1.15057	0.858945	1.315046	0.816614	1.293162	0.933791	0.988459

SA: superior-anterior; SP: superior-posterior; IA: inferior-anterior; IP: inferior-posterior; Me: mesial; Di: distal.

## Data Availability

Data used to support the findings are available from the authors upon request.
